# Identification of *HpMYB1* inducing anthocyanin accumulation in *Hippeastrum Hybridum* tepals by RNA-seq

**DOI:** 10.1186/s12870-023-04582-4

**Published:** 2023-11-28

**Authors:** Ji Li, Kunlin Wu, Lin Li, Guohua Ma, Lin Fang, Songjun Zeng

**Affiliations:** 1 Key Laboratory of South China Agricultural Plant Molecular Analysis and Gene Improvement, South China Botanical Garden, Chinese Academy of Sciences, 510650 Guangzhou, China; 2 University of Chinese Academy of Sciences, 100049 Beijing, China; 3 Guangdong Provincial Key Laboratory of Applied Botany, South China Botanical Garden, Chinese Academy of Sciences, 510650 Guangzhou, China; 4 Center of Economic Botany, Core Botanical Gardens, Chinese Academy of Sciences, 510650 Guangzhou, China

**Keywords:** Anthocyanins, Transcriptome, *Hippeastrum* × *Hybridum*, Tepal color, R2R3-MYB

## Abstract

**Background:**

Cultivated *Hippeastrum* × *hybridum* is a popular ornamental plant with large and colorful flowers, long flowering duration, and high commercial value. As its main ornamental feature, its flower color is related to the anthocyanin content in the tepals. However, the molecular regulatory mechanisms of anthocyanin biosynthesis in *H*. × *hybridum* have not yet been elucidated.

**Results:**

In the present study, 12 cDNA libraries of four stages of *H.*× *hybridum* ‘Royal Velvet’ tepal development were used for RNA-seq, obtaining 79.83 gigabases (GB) of clean data. The data were assembled into 148,453 unigenes, and 11,262 differentially expressed genes were identified. Forty key enzymes participating in anthocyanin biosynthesis were investigated, and the results showed that most of the anthocyanin structural genes were expressed at low levels in S1 and were markedly upregulated in S2 and S3. The expression profiles of 12 selected genes were verified by qRT-PCR. Furthermore, the R2R3-MYB transcription factor (TF), *HpMYB1*, involved in the regulation of anthocyanin biosynthesis was identified by sequence, expression pattern, and subcellular localization analyses. Its overexpression in tobacco significantly increased the anthocyanin levels in various tissues and activated anthocyanin-related genes.

**Conclusions:**

Using RNA-seq technology, we successfully identified a potential R2R3-MYB gene, *HpMYB1*, that regulates anthocyanin biosynthesis in *H.*× *hybridum* ‘Royal Velvet’. Our findings provide basic transcript information and valuable transcriptome data for further identification of key genes involved in anthocyanin biosynthesis and can be applied in the artificial breeding of new *H*. × *hybridum* cultivars with enhanced ornamental value.

**Supplementary Information:**

The online version contains supplementary material available at 10.1186/s12870-023-04582-4.

## Introduction

Flower color is a key characteristic of ornamental crops influenced by the type and content of anthocyanins [1]. Anthocyanins are important secondary metabolites responsible for the purple, blue, and pink colorations of various plant tissues [2]. Nearly 700 anthocyanins have been identified in nature [3], with the six most common being pelargonidin, cyanidin, delphinidin, peonidin, petunidin, and malvidin [4]. Anthocyanins can function as signals to attract pollinators as well as barriers to protect plants from damage, UV rays, and pathogens, and they respond to biotic or abiotic stress [5]. Moreover, anthocyanins act as natural antioxidants and protect against cancer and neuronal or cardiovascular diseases [6, 7]. Because of their numerous benefits, they have been a research hotspot in plant and food sciences.

Anthocyanins are synthesized through the flavonoid pathway, and their structural genes have been extensively studied in many model plants, such as *Arabidopsis thaliana*, *Petunia hybrid*, and *Zea mays* [8–10]. The structural genes encoding multiple enzymes in anthocyanin biosynthesis include chalcone synthase (CHS), chalcone isomerase (CHI), flavonoid-3-hydroxylase (F3H), flavonoid-3’-hydroxylase (F3’H), flavonoid-3’, 5’-hydroxylase (F3’5’H), dihydroflavonol 4-reductase (DFR), and anthocyanidin synthase (ANS) [4, 8]. In plant cells, the synthesized hydrosoluble anthocyanins are transported to the vacuoles for long-term stable storage [11].

In plants, the spatial and temporal expression of anthocyanin structural genes is largely regulated by R2R3-MYB, basic helix-loop-helix (bHLH), and WD repeat (WDR) transcription factors (TFs) at the transcriptional level [12, 13], among these, R2R3-MYB TFs are known to be the key regulators of anthocyanin accumulation and tissue coloration and have been identified in model plants. The first R2R3-MYB TF found to regulate anthocyanin biosynthesis was *C1*. It specifically regulates the expression of *CHS* and *DFR* genes in the aleurone layer of maize [14, 15]. Overexpression of *PAP1* upregulates the expression of *CHS*, *CHI*, and *ANS* and significantly increases the content of cyanidin in *Arabidopsis* [16]. *MYB113* and *MYB114* also participate in regulating anthocyanin biosynthesis in *Arabidopsis* [17]. The specific expression of *NtAN2* in *Nicotiana tabacum* not only activates the expression of *CHS* and *DFR* genes, but also co-regulates anthocyanin accumulation with bHLH TF [18]. In addition, similar regulatory systems involved in the accumulation of anthocyanins in ornamental plants have also been identified, including *LhMYB6* and *LhMYB12* in *Lilium* spp. [19], *AaMYB2* in *Anthurium andraeanum* [20], *PeMYB2*, *PeMYB11*, and *PeMYB12* in *Phalaenopsis* spp. [21], *GMYB10* in *Gerbera hybrida* [22], *AcMYB1* in *Aglaonema commutatum* [23], *PsMYB30* and *PsMYB58* in tree peony [24, 25], and *FhPAP1* in *Freesia hybrida* [26].

The genus *Hippeastrum*, commonly known as amaryllis, is a perennial herbaceous bulbous plants belonging to the family Amaryllidaceae [27]. Cultivated *Hippeastrum* × *hybridum* is commercially exploited as an ornamental plant mainly used for Christmas and New Year decorations worldwide because of its large and colorful flowers, long flowering duration, and upright plant architecture [28, 29]. Currently, relevant research on this genus has mainly focused on tissue culture [30], crossbreeding [31], functional components [32], and genetic diversity [33]. *H*. × *hybridum ‘*Royal Velvet’ is a single-petal flower variety with dark red tepals, which expresses the strongest red petal color and has the highest content of total anthocyanins among the six *H*. × *hybridum* cultivars [34]. Therefore, it is an ideal plant material for studying anthocyanin accumulation and the regulatory mechanism of flower coloration in *Hippeastrum*.

To date, the molecular mechanism of anthocyanin biosynthesis and its effects on tepal coloration in *Hippeastrum* remain unknown because of the lack of reference genome information and high heterozygosity. In the present study, we performed transcriptome sequencing to obtain a global view of the gene expression patterns during the four developmental stages of ‘Royal Velvet’ tepals. A novel R2R3-MYB gene, *HpMYB1*, was identified by RNA-seq analysis and functionally characterized by overexpression in tobacco. Our results improved our understanding of the potential mechanisms of tepal anthocyanin accumulation and can serve as a basis for further commercial improvement, resource utilization, and molecular breeding of *Hippeastrum* species.

## Results

### Phenotypes of tepals at different developmental stages

**Fig. 1:**
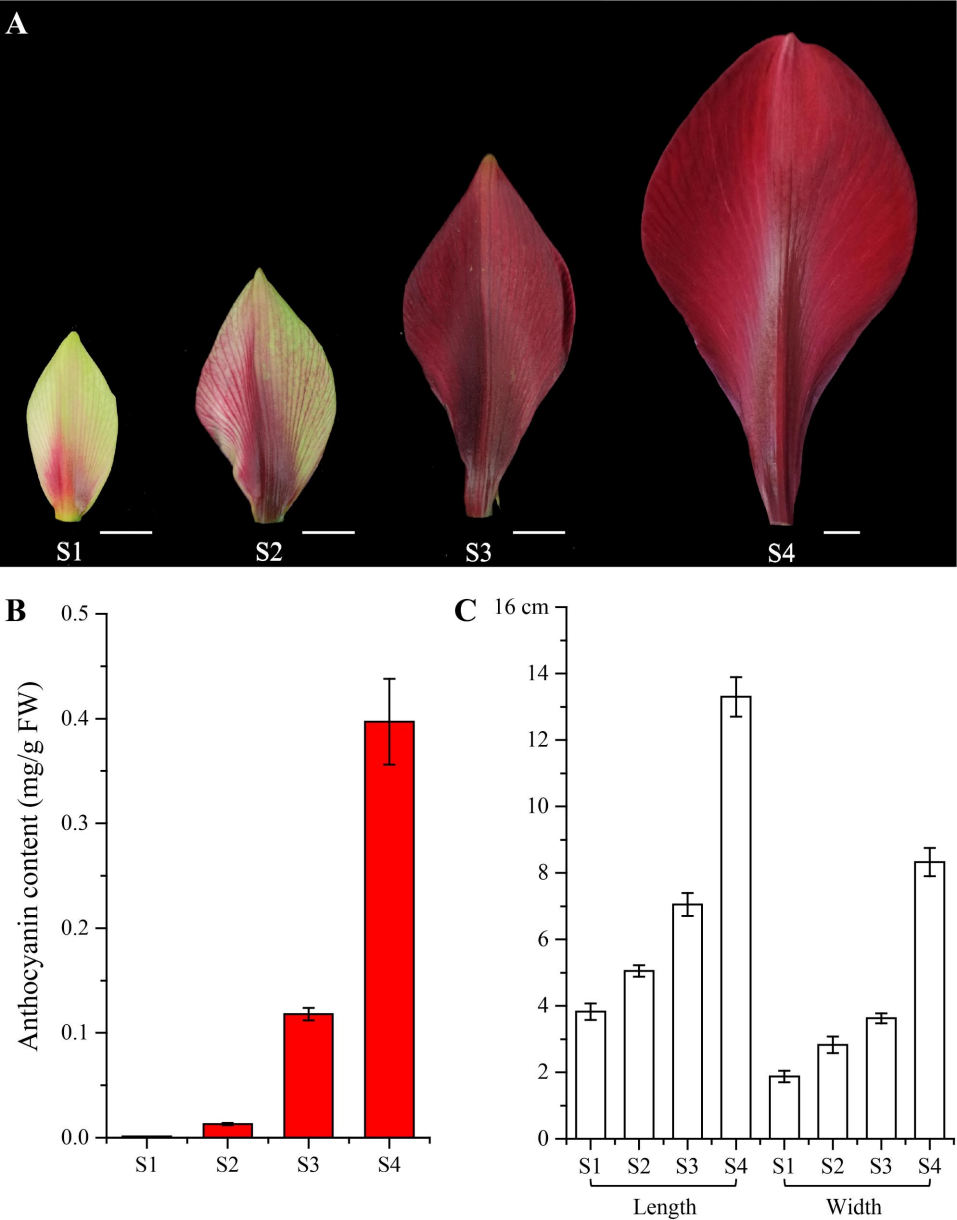
Phenotypes of tepals at different developmental stages. (**A**) The four stages of *Hippeastrum hybridum* ‘Royal Velvet’ tepals. Bar = 1 cm. (**B**) The anthocyanin content of tepals at four stages. (**C**) The length and width of tepals at four stages

The four stages of ‘Royal Velvet’ tepal development were analyzed in this study. The tepal color gradually deepened from S1 to S3 and appeared dark red in S3, indicating high accumulation of anthocyanins. In S4, the flowers were in full bloom and remained deep red (Fig. 1A). Moreover, the anthocyanin content and tepal size increased sharply from S1 to S4 and peaked at S4 (Fig. 1B, C).

### Sequencing summary, assembly, and unigene annotation

**Fig. 2:**
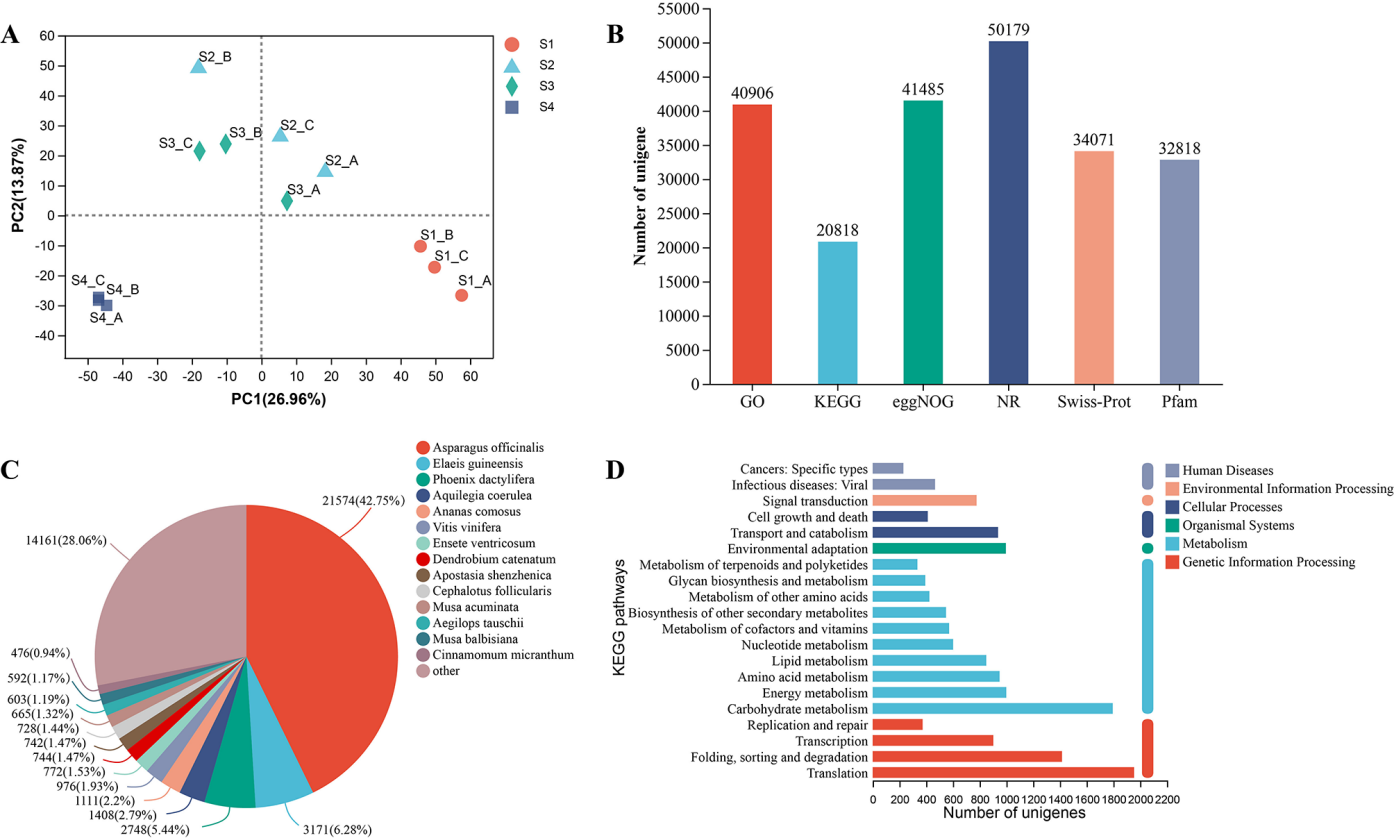
Overview of RNA-seq results and unigene annotations. (**A**) Principal components analysis of unigene expression in *Hippeastrum hybridum* ‘Royal Velvet’ tepals. (**B**) Functionl annotation of unigenes in six public database. (**C**) Species distribution of unigenes in the NR database. (**D**) The unigenes functional classifcation in KEGG databases

To elucidate the transcriptional mechanism of anthocyanin accumulation in ‘Royal Velvet,’ the RNA samples obtained from twelve tepal samples were used for transcriptome sequencing with three replicates per development stage. After the low-quality reads were filtered out, a total of 79.83 Gb of clean data and 40,753,960–51,702,956 clean reads were obtained. The Q30 value was > 93.11%. (Supplementary Table S1). The Trinity method was used to assemble 148,453 unigenes with a mean length of 739 bp and an N50 of 1116 bp (Supplementary Table S2). Among them, 138,247 (93.13%) unigenes ranged from 200 to 2000 bp in length, and 10,206 unigenes (6.87%) were over 2000 bp in length (Supplementary Table S3). Principal component analysis (PCA) revealed good repeatability between samples, making them suitable for further analysis (Fig. 2A). The unigenes were then subjected to BLAST search against six public databases to determine their potential functions. As a result, 51,769 unigenes (34.87%) were successfully annotated to at least one of the six public databases (Fig. 2B), and the 14 top-hit species based on the NR annotation were listed (Fig. 2C). The KEGG pathway enrichment analysis showed that ‘Translation,’ ‘Carbohydrate metabolism,’ and ‘Folding, sorting and degradation’ were the most significantly enriched categories (Fig. 2D).

### Differentially expressed genes (DEGs) analyses

**Fig. 3:**
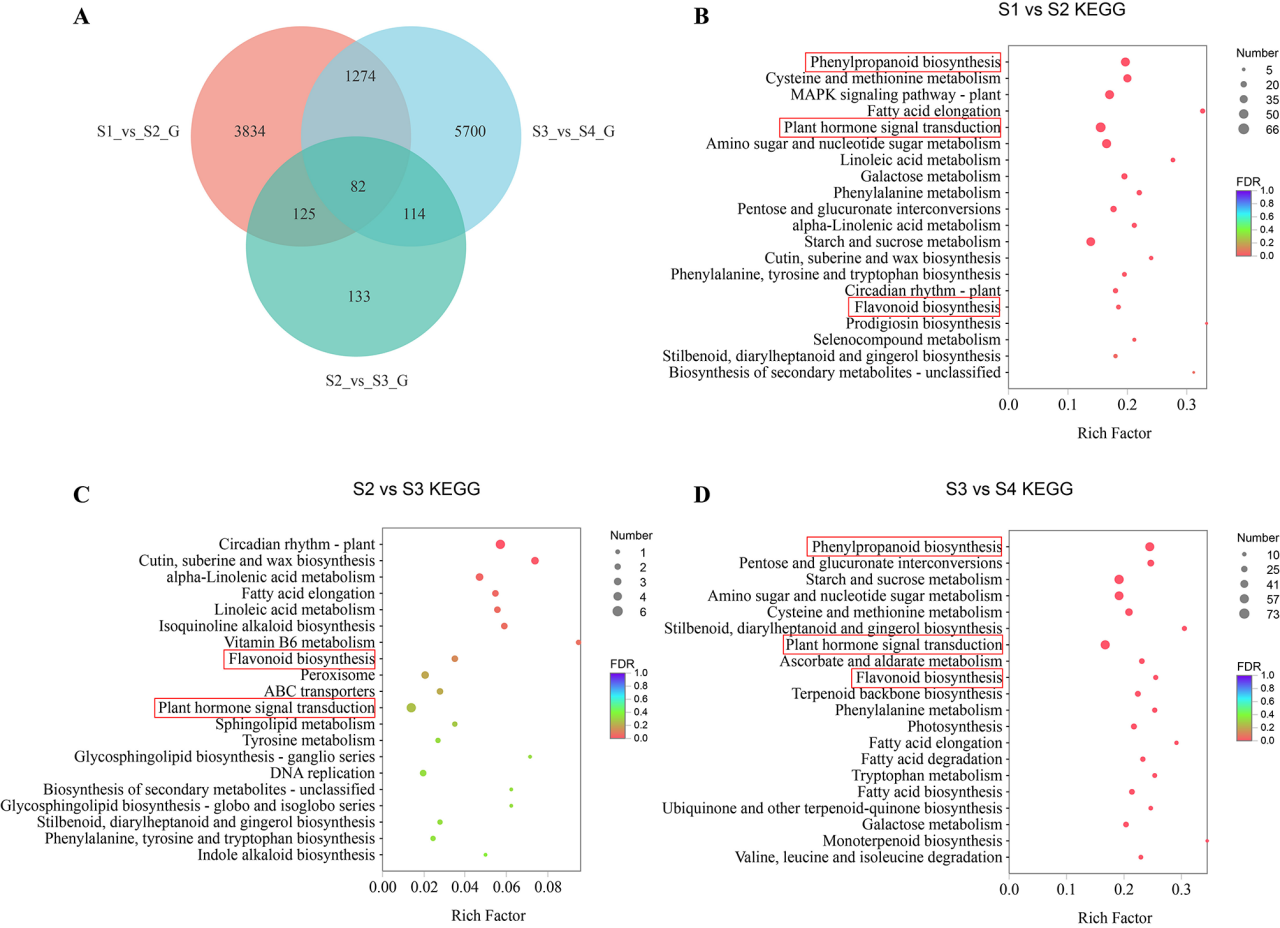
Venn diagram and functional analysis of DEGs in *Hippeastrum hybridum* ‘Royal Velvet’. (**A**) Venn diagram analysis of DEGs per comparison. (**B**-**D**) KEGG enrichment of DEGs per comparison. The red boxes indicate key pathways that may participated in pigment accumulation

To identify the DEGs involved in ‘Royal Velvet’ tepal coloration, the TPM values were analyzed at the four developmental stages (FDR ≤ 0.05 and fold change ≥ 2). A total of 11,262 DEGs were identified through pairwise comparisons, of which 5315, 454, and 7170 DEGs were found between S1 vs. S2, S2 vs. S3, and S3 vs. S4, respectively. A Venn diagram analysis of the DEGs indicated that 82 genes were shared among all three comparisons (Fig. 3A). KEGG enrichment analysis was performed to determine the functionality of the identified DEGs. The top 20 enriched KEGG pathways of the unigenes are shown in Fig. 3B–D. Among them, ‘Phenylpropanoid biosynthesis,’ ‘Plant hormone signal transduction,’ and ‘Flavonoid biosynthesis’ were the key pathways involved in pigment accumulation. Besides, DEGs analysis comparing S2, S3, and S4 with S1 and GO enrichment analysis of DEGs were also provided in Supplementary Fig. S2 and S3.

### The anthocyanin biosynthesis pathway

**Fig. 4:**
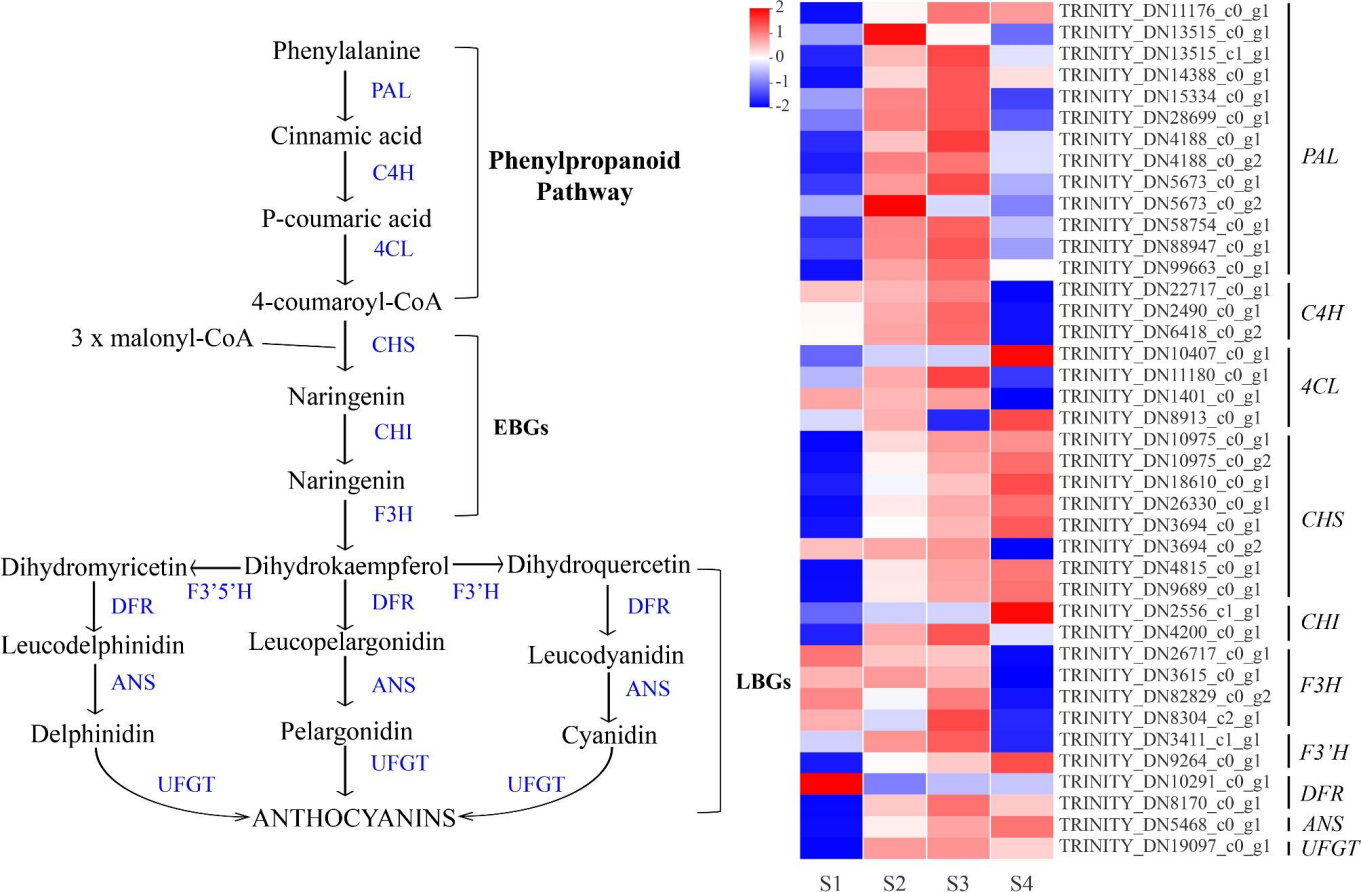
Heatmap constructed using anthocyanin biosynthesis related unigenes. The heatmap is generated based on the mean expression levels of anthocyanin structural gene using the TPM values from RNA-seq. Blue represents low expression levels, while red signifies high expression levels. EBGs: early biosynthesis genes; LBGs: late biosynthesis genes; PAL: phenylalanine ammonia-lyase; C4H: trans-cinnamate 4-monooxygenase; 4CL: 4-coumarate-CoA ligase 2; CHS: chalcone synthase; CHI: Chalcone isomerase; F3H: Flavanone 3-hydroxylase; F3’H: favanone 3-hydroxylase; F3’5’H: flavonoid 3’, 5’- hydroxylase; DFR: dihydrofavonol 4-reductase; ANS: anthocyanidin synthase; UFGT: UDP-favonoid glucosyl transferase

Anthocyanin accumulation is one of the most important factors affecting tepal color, especially in red flowers [2]. In the present study, 40 unigenes responsible for anthocyanin biosynthesis were isolated, and expression heatmaps were constructed based on the TPM values (Fig. 4). Thirteen PAL, three C4H, and four 4CL upstream genes involved in anthocyanin biosynthesis were identified, most of which were upregulated in S2 and S3 compared to those in S1 and S4. Seven of the eight *CHS* unigenes were upregulated continuously from S1 to S4, whereas the expression level of all *F3H* unigenes in S4 was lower than that in the other three stages. The predicted unigenes included two *F3’H*, two *DFR*, one *ANS*, and one *UFGT* were screened as late biosynthesis genes (LBGs), and most of them (except for TRINITY_ DN10291_c0_g1 and TRINITY_DN3411_c1_g1) were down-regulated in S1 compared with those in S2 to S4. The detailed TPM values for each unigene are provided in Supplementary Table S4.

**Fig. 5:**
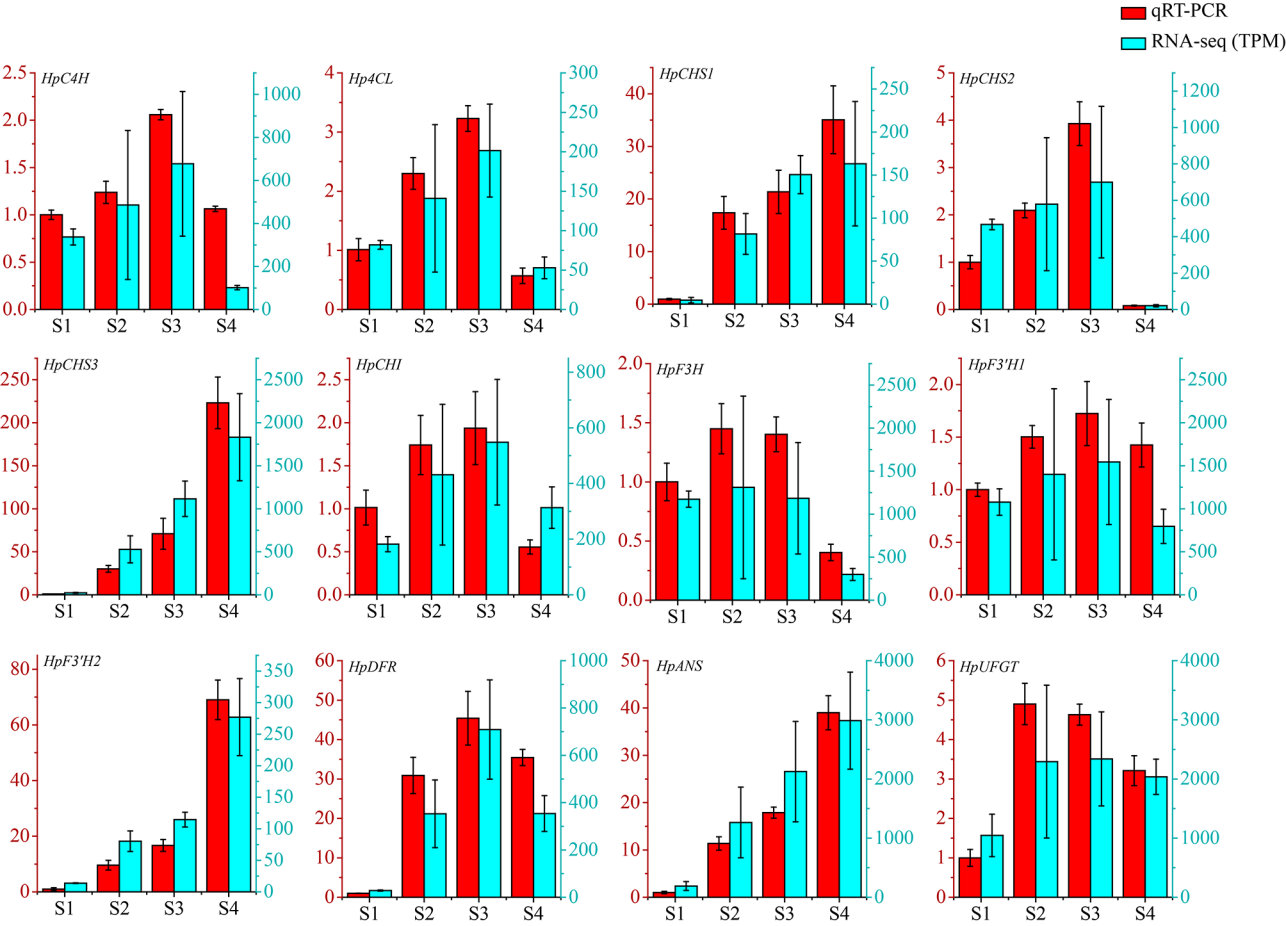
qRT-PCR verifcation of the expression profles of selected unigenes involved in anthocyanin biosynthesis. Twelve unigenes from the anthocyanin biosynthesis pathway were used to validate the RNA-seq results. The expression levels were calculated by the 2^−∆∆CT^ method. The y-axis on the left represents the unigene relative expression by qRT-PCR and the y-axis on the right indicates the TPM value of the unigene in RNA-seq data. The unigene ID and specific primers were provided in Supplementary Table S5

To validate the credibility of the transcriptome sequencing data, 12 unigenes were selected from the anthocyanin biosynthesis pathway, and their expression levels were analyzed at four stages using qRT-PCR (Fig. 5). Our qRT-PCR results were consistent with those obtained using the RNA-seq method, indicating that the use of RNA-seq for counting reads was reliable for further analysis.

### Identification of key TFs in regulating anthocyanin biosynthesis in the tepals of *H. × hybridum* ‘Royal Velvet’

**Fig. 6:**
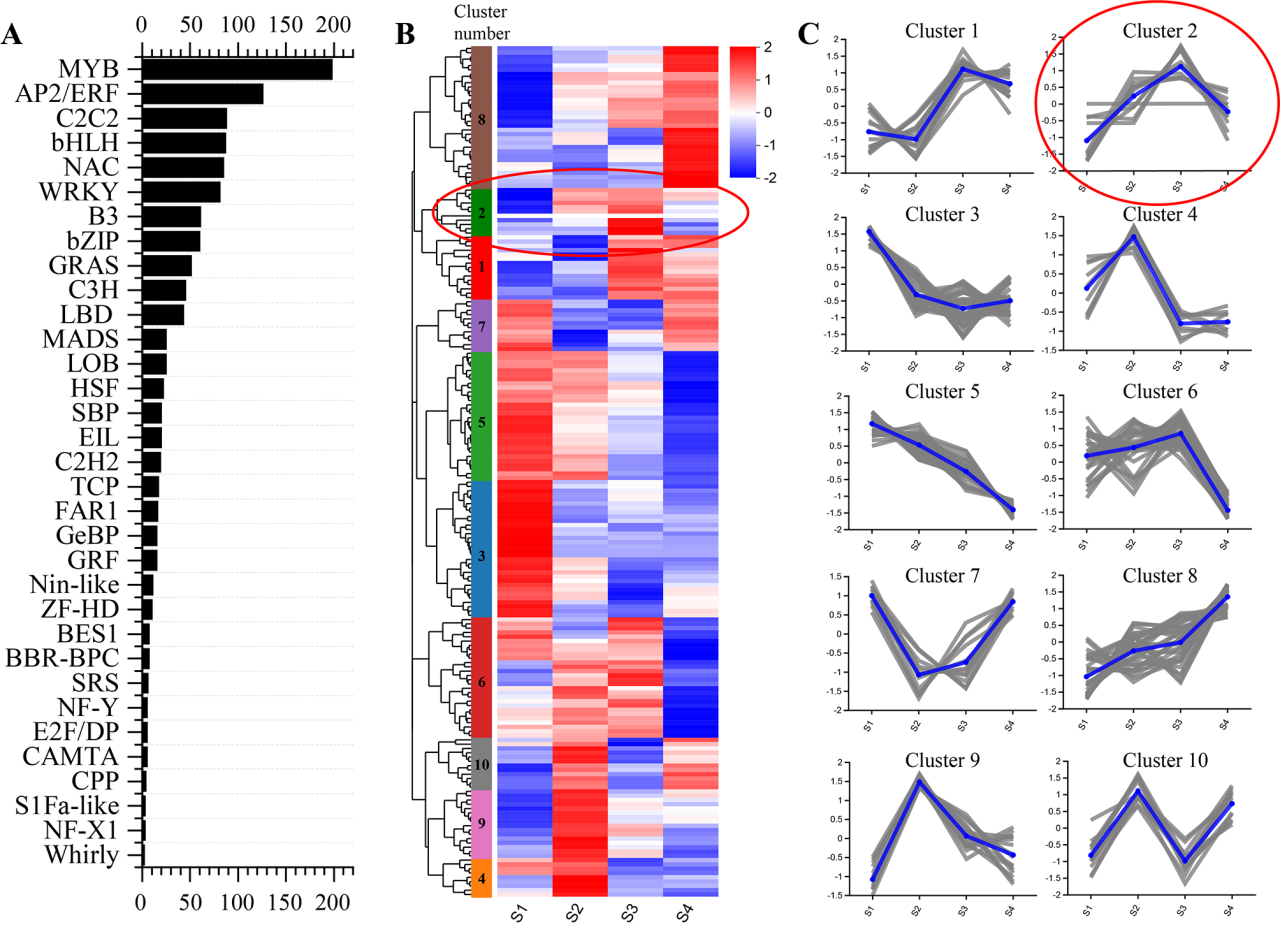
Analysis of transcription factors based on transcriptome sequencing. (**A**) Top 20 transcription factor families in *Hippeastrum hybridum* ‘Royal Velvet’ tepals. (**B**) Heatmaps showing the expression profile of 198 MYB, based on standardized TPM values. Ten different color modules represent different clusters and cluster 2 was circled in a red oval. (**C**) 198 MYB were grouped into ten clusters depending on the expression profile. the y-axis represent the normalized expression

Transcription factors (TFs) play a major role in color formation by regulating the expression of key enzymes involved in anthocyanin biosynthesis. In our study, 1187 TFs belonging to 33 families were identified and classified using RNA-seq annotation. MYB (198), AP2/ERF (126), and C2C2 (88) were the top three TF families by number (Fig. 6A).

Furthermore, MYB TFs were studied to deepen our understanding of their involvement in the regulation of dark red color formation at the four developmental stages of ‘Royal Velvet’ tepals. Heatmaps of the 198 MYB TFs were constructed and the TFs were divided into ten clusters based on their expression profiles (Fig. 6B and C). The results showed that the expression patterns of MYB TFs in cluster 2 were significantly positively correlated with the changes in most anthocyanin structural genes at the four developmental stages. Furthermore, the expression patterns and sequence homology of the 11 unigenes from cluster 2 were analyzed, and one unigene with ID TRINITY_DN4295_c0_g1 had an R2R3 DNA-binding domain and high homology with known positive anthocyanin regulators in other plants. Thus, this unigene was selected as a potential MYB TF involved in the regulation of anthocyanin biosynthesis to be investigated in the subsequent study.

### Isolation and sequence analysis of *HpMYB1*

**Fig. 7:**
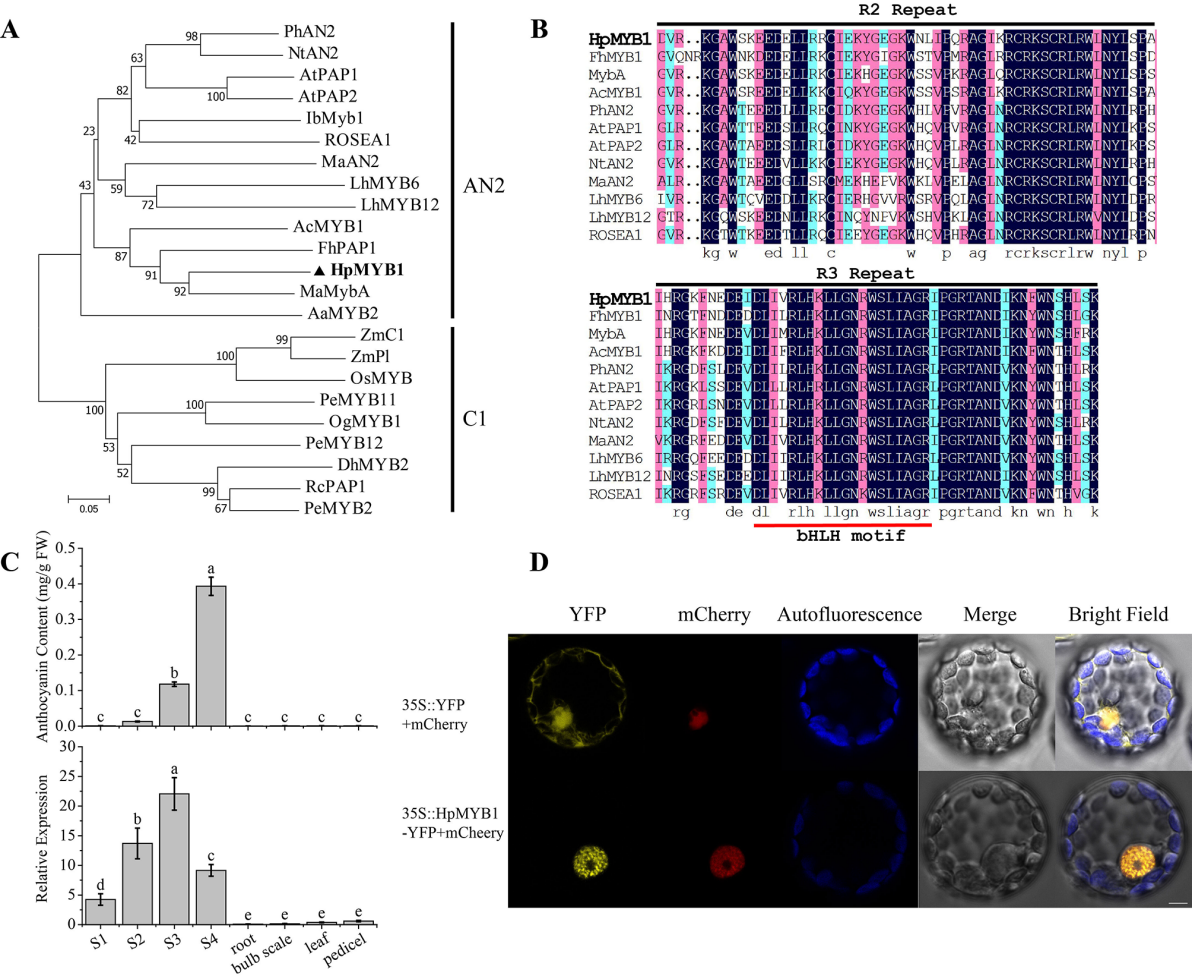
Sequence, expression patterns and subcellular localization analysis of HpMYB1. (**A**) Phylogenetic tree of HpMYB1 and 22 other known anthocyanin-related R2R3-MYBs in other plants. (**B**) Multiple alignment of the conserved R2-domain and R3-domain of different anthocyanin biosynthesis related R2R3-MYB proteins. The black line showed the conserved R2 and R3 domain, The red line indicated a motif ([D/E]Lx_2_[R/K]x_3_Lx_6_Lx_3_R) interacting with a bHLH TF. (**C**) The anthocyanin content and the relative expression of *HpMYB1* at different tepal developmental stages and different tissues. (**D**) Subcellular localization of HpMYB1 protein in *Arabidopsis thaliana* mesophyll protoplasts. Bar = 5 μm, YFP: yellow fluorescence protein; mCherry: nuclear localization

Based on the RNA-seq data, the sequences of the unigene TRINITY_DN4295_c0_g1 from the tepals of ‘Royal Velvet’ were cloned by PCR and designated as *HpMYB1* (GenBank accession number: OQ446482). *HpMYB1* has a 696-bp open reading frame (ORF) and encodes 231 amino acids. A bootstrapped phylogenetic tree generated using MEGA 7.0 with the neighbor-joining method showed that HpMYB1 clustered with AcMYB1, FhPAP1, and MybA in the AN2 subgroup (Fig. 7A). Multiple amino acid sequence alignment of HpMYB1 and other known positive anthocyanin regulators showed that the conserved R2 and R3 domains were located at the N-terminus, and a conserved bHLH motif interacting with a bHLH TF was located in the R3 domain (Fig. 7B). The GenBank accession numbers from this study are provided in Supplementary Table S6.

### Expression patterns and subcellular localization of *HpMYB1*

The transcript levels of *HpMYB1* and anthocyanin concentration in the four stages of tepal development and in different tissues of ‘Royal Velvet’ were monitored. The results showed that *HpMYB1* mRNA was highly expressed in the tepals at all four developmental stages, in which anthocyanins were highly accumulated, whereas no or trace amounts of *HpMYB1* mRNA were detected in the tissues with no anthocyanin accumulation, such as roots, bulb scales, leaves, or pedicels (Fig. 7C). These results strongly suggested that *HpMYB1* expression was correlated to the anthocyanin accumulation in ‘Royal Velvet.’

To function as a regulator, HpMYB1 must be located inside the nucleus of plant cells. Thus, subcellular localization analysis of the HpMYB1 protein was performed. HpMYB1 was fused to a yellow fluorescent protein (YFP) and cloned into the pSAT6-EYFP-N1 vector. The YFP fluorescent signal of recombinant 35 S::HpMYB1-YFP was detected in the nucleus, similar to the nuclear localization marker mCherry (Fig. 7D). These results confirmed that HpMYB1 is localized in the nucleus.

### Effects of HpMYB1 overexpression in Tobacco

**Fig. 8:**
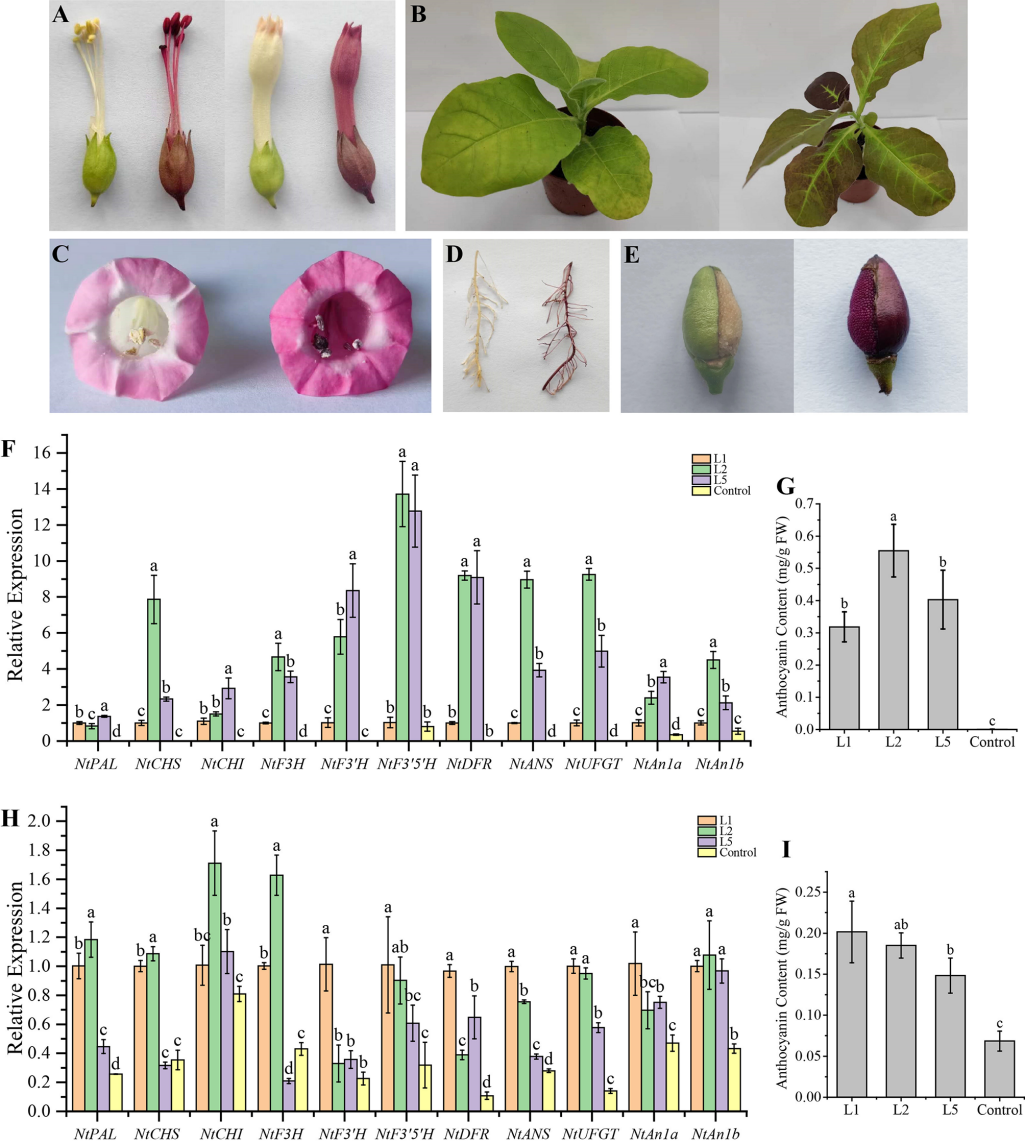
Overexpression of*HpMYB1*strongly promoted anthocyanin accumulation in tobacco. (**A**) Flower, filaments, anthers, stigma and sepal phenotypes of overexpressing (OE)-*HpMYB1* line (left) and control (right). (**B**) Leaf phenotypes of OE-*HpMYB1* line and control. (C) Corolla phenotypes of OE-*HpMYB1* line and control. (**D**) Root phenotypes of OE-*HpMYB1* line and control. (**E**) Ovary wall and seed coat phenotypes of OE-*HpMYB1* line and control. (**F**) and (**G**) represent the relative expression profles of anthocyanin-related genes and anthocyanin content of the OE-*HpMYB1* lines, respectively. (**H**) and (**I**) represent the relative expression profles of anthocyanin-related genes and anthocyanin content of the control plant respectively. L1, L2, and L5 mean three transgenic tobacco lines of OE-*HpMYB1* and the *NtActin* were used as the reference gene

To characterize the role of HpMYB1 in the regulation of anthocyanin biosynthesis, *HpMYB1* driven by the constitutive CaMV 35 S promoter was introduced into tobacco leaf discs via *Agrobacterium*-mediated transformation, and significant phenotypic changes in both vegetative and reproductive tissues were observed in the transgenic lines. In comparison with the reproductive tissues of the wild type, darker corollas, pigmented filaments, anthers, pistils, calyces, ovaries, and seed coats were observed in the HpMYB1 overexpression lines (Fig. 8A, C, and E). Similarly, the leaves and roots conferred an intense red-purple color upon *HpMYB1* overexpression tobacco (Fig. 8B, D). The total anthocyanin content in the leaves and corollas of the *HpMYB1* transgenic lines was higher than that of the control (Fig. 8G and I).

To confirm the target genes of HpMYB1, a qRT-PCR analysis was performed on the leaves and corollas of both transgenic tobacco and control plants to analyze the expression levels of genes involved in the anthocyanin pathway, including nine structural genes and two bHLH TF regulators. The results indicated that all 11 genes related to anthocyanin biosynthesis were markedly upregulated in the leaves of all three transgenic lines compared to those in the control (Fig. 8F). Likewise, the transcript abundances of these 11 anthocyanin-specific genes were significantly higher in the corollas of the three transgenic lines than in those of the control (Fig. 8H). These results suggested that HpMYB1 is a functional MYB TF factor that regulates anthocyanin biosynthesis.

## Discussion

### Application of transcriptome sequencing for studying the anthocyanin regulation mechanism of *H. × hybridum* ‘Royal Velvet’ tepals

*H*. × *hybridum*, a species of the genus *Hippeastrum*, has high ornamental value and is popular worldwide [28, 29]. For nearly 200 years, breeders have committed themselves to cultivating new *Hippeastrum* hybrid species [31]. However, the lack of reference genome data and high heterozygosity have greatly limited the research on the molecular mechanisms of key trait formation in this genus. Transcriptome sequencing provides a low-cost, rapid, and reliable method to monitor gene expression patterns and identify key genes at various developmental stages or under different physiological conditions in non-model plants lacking genomic information [35, 36]. In grape hyacinth, transcriptome sequencing of the blue and white variants provided a foundation for future functional and molecular biological studies on flower color [37]. In *Dendrobium nestor*, a transcriptomic analysis of petal samples from three developmental stages helped discover the key candidate genes of the anthocyanin pathway and revealed the mechanism of purple color formation [38]. In the present study, we applied the transcriptome sequencing technology to perform a global transcript analysis of the four tepal development stages of *H.* × *hybridum* ‘Royal Velvet’ and investigate the molecular mechanism underlying the anthocyanin regulation and dark red flower color formation in this species. Finally, we annotated 148,453 unigenes with an annotation rate of 34.87% to at least one of the six public databases, which was similar to those of other *H. × hybridum* [33, 39] and *Amaryllidaceae* species [40, 41]. Our transcriptome sequencing also provided global gene expression data for further studies on *H. × hybridum* ‘Royal Velvet.’

### Floral color and key structural genes in the anthocyanin biosynthesis of *H. × hybridum* ‘Royal Velvet’

In *H*. × *hybridum*, flowers provide visual attractiveness, and the commercial value of ornamental plants is largely related to their tepals. Anthocyanins are the major pigments that confer red, blue, and purple coloration to various plant tissues [42, 43]. Accumulation of anthocyanins can be closely associated with flower color. In *Hydrangea macrophylla*, the infertile blue flower color is mainly correlated with an increase in anthocyanin content [44]. In buckwheat, higher anthocyanin accumulation was detected in the cotyledons and flowers of the red cultivar than in those of the white cultivar at different growth stages [45]. A similar phenomenon has been reported in azalea [46], *Camellia sinensis* [47], and pagoda trees [48]. In the present study, the anthocyanin content in the tepals of ‘Royal Velvet’ at four flowering stages was determined for the first time. With the rapid increase in total anthocyanin levels from S1 to S4, the tepals finally turned dark red when the flowers opened.

Anthocyanin biosynthesis has always been a research hotspot of plant secondary metabolism research, and the corresponding genes include *PAL*, *C4H*, *4CL*, *CHS*, *CHI*, *F3H*, *F3’H*, *DFR*, *ANS*, and *UFGT* [1, 49]. Anthocyanin pathway genes play important roles in flower coloration [50, 51]. Chalcone synthase encoded by the *CHS* gene is a core enzyme involved in anthocyanin biosynthesis, and decreasing the expression of *CHS* can lead to the fading of flower color [52]. Using RNA interference technology to reduce the transcript level of *F3H* gene in summer viola, the flower color changed from blue-purple to white [53]. Similar results have been reported for calli [54], morning glories [55], and *Phalaenopsis amabilis* [56]. In ‘Royal Velvet,’ most of the anthocyanin biosynthesis enzyme genes identified from transcriptome data were at a low expression level in S1 and markedly upregulated at S2 and S3 (Fig. 4), displaying a strong correlation between their mRNA levels and anthocyanin concentration in the tepals. The anthocyanin content reached its peak and resulted in a dark red color in S4, when the expression levels of seven *CHS* (DN10975_c0_g1, DN10975_c0_g2, DN18610_c0_g1, DN26330_c0_g1, DN3694_c0_g1, DN4815_c0_g1, DN9689_c0_ g1), one *CHI* (DN2556_c1_g1), one *F3’H* (DN9264_c0_g1), one *DFR* (DN8170_c0_g1), one *ANS* (DN5468_c0_g1), and one *UFGT* (DN19097_c0_g1) were high, suggesting their important roles in the coloration of mature tepals.

### HpMYB1 is a functional R2R3-MYB TF involved in regulating anthocyanin biosynthesis

It has been revealed that the binding of R2R3-MYB TFs to the promoter of anthocyanin structural genes strongly induces their expression and anthocyanin accumulation [18, 57, 58]. In apples, *MdMYB1* can act as an activator to regulate the expression of anthocyanin pathway genes and determine skin color [59]. Overexpression of an anthocyanin-promoting R2R3-MYB TF, GMYB10, significantly increased the anthocyanin content and induced the production of new anthocyanins in *Gerbera hybrida* [22, 60]. In the present study, we found that HpMYB1 contains a conserved bHLH motif in the R3 domain (Fig. 7B), suggesting that the combination of HpMYB1 with bHLH TFs co-regulates anthocyanin biosynthesis, which has been reported in many other plant species [13, 61]. Our phylogenetic analysis showed that HpMYB1 is an anthocyanin-specific MYB activator in the AN2 subgroup and is closely related to MaMybA (Fig. 7A), a known anthocyanin-related R2R3-MYB TF isolated from *Muscari armeniacum* flowers [62]. The gene expression profiles of *HpMYB1* were in accordance with those of most anthocyanin biosynthetic genes in ‘Royal Velvet,’ such as *HpC4H*, *Hp4CL*, *HpCHS2*, *HpCHI*, *HpF3H*, *HpF3’H1*, *HpDF*R, and *HpUFGT* (Fig. 5). More importantly, heterologous expression of *HpMYB1* in tobacco resulted in higher anthocyanin concentrations in both vegetative and reproductive tissues, and strong activation of anthocyanin-related genes was observed in the transgenic lines compared to those in the control plants (Fig. 8). Therefore, we hypothesized that *HpMYB1* is a R2R3-MYB transcriptional activator involved in regulating anthocyanin biosynthesis in ‘Royal Velvet’ tepals. Functional verification of *HpMYB1* in *H.* × *hybridum* calli was also performed (data not shown).

## Conclusions

In this study, a de novo assembly of transcriptome data was performed to provide preliminary insights into the mechanisms of anthocyanin accumulation in the four stages of ‘Royal Velvet’ tepal development. The unigene expression pattern analysis revealed that anthocyanin biosynthesis-related genes were significantly upregulated in S2 and S3. An R2R3-MYB TF, *HpMYB1*, involved in anthocyanin biosynthesis regulation, was identified by RNA-seq analysis, and its mRNA profiles were in accordance with those of most anthocyanin structural genes and verified by qRT-PCR analysis. Furthermore, the stable genetic transformation of *HpMYB1* strongly increased the pigment levels in various tissues and activated anthocyanin-related genes in tobacco. Our results lay the foundation for understanding the molecular mechanisms underlying the accumulation of anthocyanins and flower coloration in *H.*× *hybridum.*

## Materials and methods

### Plants and materials

Bulbs of *H.* × *hybridum* ‘Royal Velvet’ with a circumference of 30–32 cm were purchased from Guangdong Shengyin Flower and Gardening Co., LTD. They were stored at 4 ± 1℃ for about 45 days to break dormancy. Subsequently, they were planted in the greenhouse of the South China Botanical Garden, Chinese Academy of Sciences (Guangzhou, China). The flower development of ‘Royal Velvet’ can be divided into four stages: Stage 1 (S1): Flower buds are encased in bracts, almost no pigment is present (lasting for three days); Stage 2 (S2): The flower buds are just emerging from the bract, pigment begins to accumulate (lasting for six days); Stage 3 (S3): Flowers are about to bloom with obvious pigment accumulation (lasting for nine days); Stage 4 (S4): Flowers in full bloom with dark-red pigment coloration (lasting for 12 days). The phenotypes of the four flower developmental stages are shown in Supplementary Fig. S1. Tepal tissue samples were collected between 10 a.m. and 11 a.m. on May 2020. Tobacco (*Nicotiana tabacum* ‘NC89’) used for subsequent experiments was cultivated in a long-day phytotron (16 h/8 h light/dark, 25 °C/18°C). The samples were immediately frozen in liquid nitrogen and stored at -80 °C until further analyses.

### Anthocyanin content determination

The tissues of ‘Royal Velvet’ and tobacco were ground into powder by ball milling, and total anthocyanins were extracted using the pH difference method [63]. Briefly, the powder (0.1 g) was mixed with 2 mL of methanol (containing 0.05% HCl) overnight, after which KCl (0.025 M, pH 1.0) and NaAc (0.4 M, pH 4.5) solutions were used to determine the anthocyanin content. Both solutions (40 µL of each) were mixed with 160 µL aliquot for 15 min in darkness before the measurements. The absorbance at 510 and 700 nm was measured using an ELISA reader (BioTek, Winooski, USA).

### RNA preparation and transcriptome sequencing

Total RNA was extracted from ‘Royal Velvet’ and tobacco tissues using the RNAprep Pure Plant Kit (TIANGEN, China), according to the manufacturer’s instructions. Integrity and purity of the total RNA were assessed using a 1% agarose gel and 2100 Bioanalyzer (Agilent Technologies, Palo Alto, CA, USA) and quantified using a NanoDrop 2000 spectrophotometer (Thermo Fisher Scientific, Wilmington DE, USA). Only high-quality RNA samples were used to construct the sequencing library and analyze the gene expression patterns.

A total of 12 RNA samples of ‘Royal Velvet’ tepals were used to construct the sequencing libraries using the Illumina TruSeqTM RNA sample preparation kit (San Diego, CA) and sequenced on an Illumina NovaSeq 6,000 platform generating paired-end reads. The clean sequence reads were assembled *de novo* using Trinity (http://trinityrnaseq.sourceforge.net/) after filtering the low-quality reads and adaptors. Trinity grouped the transcripts into clusters according to their shared sequence content, and the longest transcript was selected as the unigene. BUSCO software (Version 3.0.2) was used to evaluate the quality of transcriptome assembly. For functional annotation, the assembled unigenes were aligned to public protein databases, including the GO, KEGG, NR, eggNOG, Pfam, and Swiss-Prot databases, using BLASTx. The transcript per million reads (TPM) value was quantified by RSEM software (Version: 1.3.1, http://deweylab.biostat.wisc.edu/rsem/) to calculate the gene expression of each unigene. Differentially expressed genes (DEGs) were identified using the R package DESeq2 for subsequent analysis, and the unigenes with FDR < 0.05 and |Log2 fold change|≥ 1 were considered DEGs. Tools and softwares used in the study were provided in Supplementary Table. S7.

### Gene cloning and sequence analysis

Total RNA from the tepals of ‘Royal Velvet’ at S3 was used to synthesize first strand cDNA using the TransScript® One-Step gDNA Removal cDNA Synthesis SuperMix (Transgen, Beijing, China). Sequences from the transcriptome data were used to design specific primers using the SnapGene software (V2.3.2). Full-length cDNA of *HpMYB1* was amplified using the Phanta Max Super-Fidelity DNA Polymerase (Vazyme Biotech Co., Ltd., Nanjing, China). The PCR products were cloned into the pEASY®-Blunt Cloning Kit (TransGen Biotech Co., Ltd.; Beijing, China) and sequenced. For the phylogenetic analysis and sequence alignment, full-length deduced amino acid sequences were analyzed using the DNAMAN (v 8.0.8.789) and MEGA (v 7.0.26) programs (neighbor-joining method with 1000 bootstrap replicates), respectively.

### Subcellular localization

A fragment of *HpMYB1* containing ORF was inserted into the *Eco*RI and *Bam*HI sites of the pSAT6-EYFP-N1 vector to create the recombinant plasmid 35 S::HpMYB1-YFP, which was driven by the CaMV 35 S promoter. The control vector 35 S::YFP, nuclear marker 35 S::mcherry, and 35 S::HpMYB1-YFP were introduced by polyethylene glycol (PEG)-mediated transient transformation [64]. After culture at 28 °C in darkness for 16 h, the protoplasts were examined under a Zeiss LSM 510 confocal microscope (Zeiss, Jena, Germany) with a cooled digital CCD camera (Zeiss, Jena, Germany) for imaging.

### qRT-PCR analysis

qRT-PCR was performed to investigate the gene expression levels in ‘Royal Velvet’ and tobacco tissues and validate the expression profile data of RNA-seq. Total RNA extraction and first-strand cDNA synthesis were performed using the kits described above. Polymerase chain reactions were conducted in a LightCycler 480 II system (Roche, Mannheim, Germany) using the PerfectStart Green qPCR SuperMix (TransGen Biotech Co., Ltd., Beijing, China). The total reaction volume was 20 µL, containing 1 µL of the cDNA template. The amplification was programmed as follows: 94℃ for 30 s, 45 cycles at 94℃ for 5 s, 60℃ for 30 s. The cycle threshold (Ct) values were calculated using the LightCycler 480 software (v1.5.1.62) and the relative gene expression was estimated with the 2^−ΔΔCT^ method [65]. *HpEF-1a*, *HpGAPDH2* [66] and *NtActin* (accession number: X69885) were used as reference genes.

### Overexpression of *HpMYB1* in Tobacco

To determine the function of HpMYB1, the ORF of *HpMYB1* without stop codons was inserted into the *Eco*R I and *Xba* I sites of the pGreen-C17 vector driven by the CaMV 35 S promoter. The recombinant plasmid pGreen-C17-HpMYB1 was transferred into *Agrobacterium tumefaciens* strain EHA105 using the freeze-thaw method and then transformed into tobacco using the leaf disc method [67]. T1-generation corollas and leaves from transgenic and control tobacco plants were collected for anthocyanin content determination and qRT-PCR analysis.

### Statistical analysis

All charts were plotted with average values and standard errors of the parameters using Origin Pro (2021) (v9.8.0.200). Statistical significance was tested by one-way ANOVA followed by Duncan’s (D) test using the SPSS software (v 25.0), with a significance level of p < 0.05. All measurements were performed in triplicates.

### Electronic supplementary material

Below is the link to the electronic supplementary material.

Supplementary Material 1

Supplementary Material 2

## Data Availability

The raw data of the 12 samples used in the present study were submitted to the NCBI Short Read Archive (SRA) under the BioProject accession number PRJNA943557.

## Abbreviations

DEGs: Differentially expressed genes
TF: Transcription factor
NR: Non-redundant protein sequence database
KEGG: Kyoto Encyclopedia of Genes and Genomes
GO: Gene Ontology
eggNOG: evolutionary genealogy of genes:Non-supervised Orthologous Groups
Pfam: Protein family
Swiss-Prot: Swiss-Prot Protein Sequence Database
qRT-PCR: Quantitative real-time polymerase chain reaction
RACE: Rapid-amplification of cDNA ends
Ct: cycle threshold
TPM: Transcript per million reads
CaMV: cauliflower mosaic virus
ORF: Open reading frame
RNA-seq: RNA sequencing
