# Supplementary material for: Identification of HpMYB1 inducing anthocyanin accumulation in Hippeastrum Hybridum tepals by RNA-seq
Source: BMC Plant Biol. 2023 Nov 28;23:594. doi: 10.1186/s12870-023-04582-4 (PMC10683291; doi:10.1186/s12870-023-04582-4)

**Supplementary figure 1.** Different flower developmental stages of ‘Royal Velvet’. Bar =1cm.


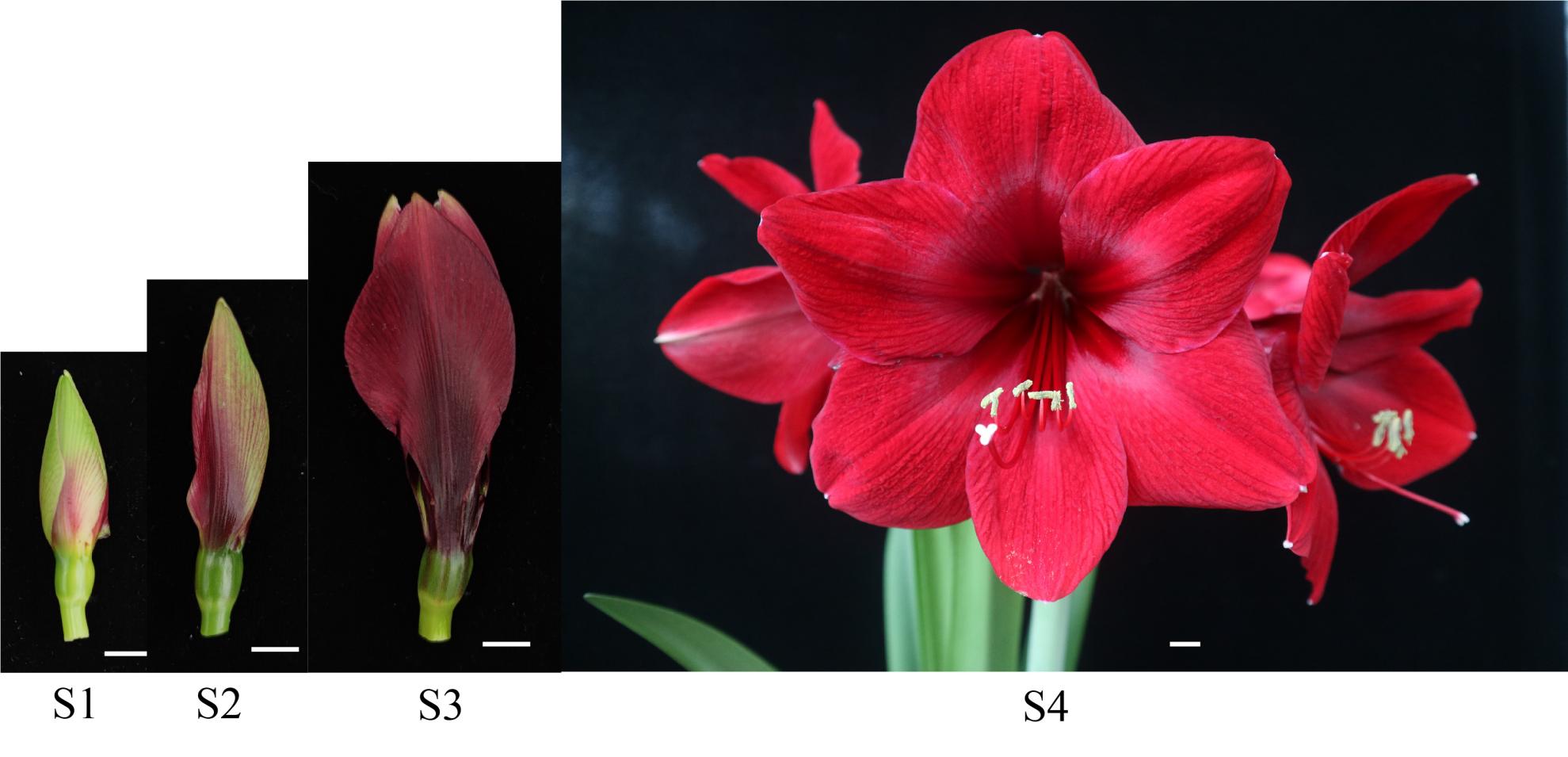


**Supplementary figure 2. DEGs analysis comparing S2, S3, and S4 with S1 in *Hippeastrum hybridum* ‘Royal Velvet’**. (A) Venn diagram analysis of DEGs per comparison. (B-D) KEGG enrichment of DEGs per comparison.


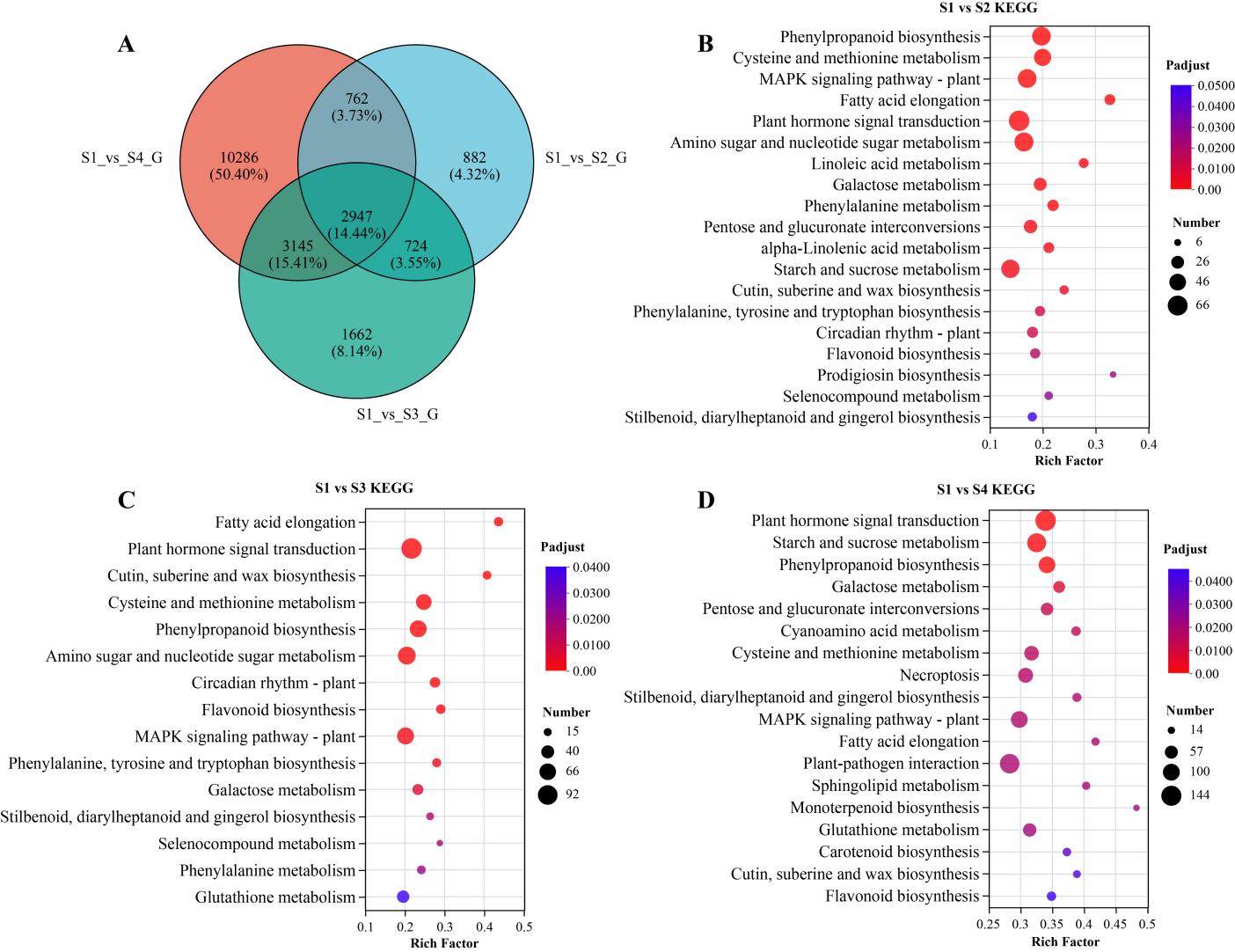


**Supplementary figure 3. GO enrichment analysis of DEGs per comparison in *Hippeastrum hybridum* ‘Royal Velvet’.**


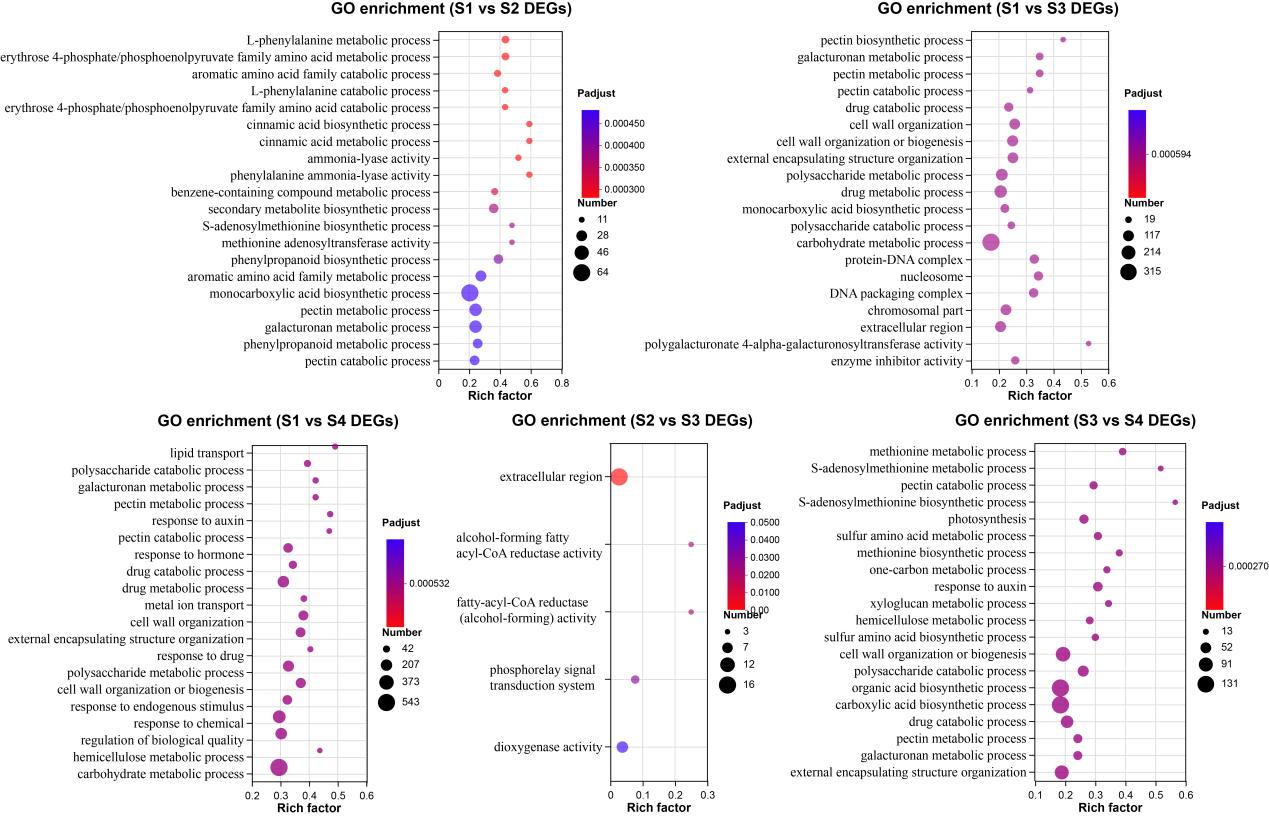

Supplement: Supplementary file 2 — Supplementary Material 2 [file 12870_2023_4582_MOESM2_ESM.docx]
